# The unprecedented avian influenza crisis in Japan: Strategies for prevention and response

**DOI:** 10.1016/j.nmni.2023.101130

**Published:** 2023-04-20

**Authors:** Yudai Kaneda, Akihiko Ozaki, Tshewang Gyeltshen, Tetsuya Tanimoto

**Affiliations:** School of Medicine, Hokkaido University, Hokkaido, Japan; Department of Breast and Thyroid Surgery, Jyoban Hospital of Tokiwa Foundation, Fukushima, Japan; Graduate School of Public Health, St. Luke's International University, Tokyo, Japan; Department of Internal Medicine, Jyoban Hospital of Tokiwa Foundation, Fukushima, Japan

**Keywords:** Avian influenza, COVID-19, Japan

“Dear Editor”

The number of confirmed positive cases of highly pathogenic avian influenza virus (HPAIV) in wild birds for the 2022 season was 187, recording the highest number in Japan's statistical history [1]. Approximately 9.98 million chickens have been subjected to culling after the rising cases of HPAIV [1]. This forms about 10% of the total industrial livestock farming in Japan. The peak of infection is still not observed, and the government has initiated a nationwide instruction for thorough emergency sanitization from December 22, 2022. Most of the reasons that make the HPAIV problematic are due to industrial damage caused by large-scale culling when the poultry is infected. Moreover, although human-to-human transmission is a rare phenomenon, increasing H5N1 and H5N6 cases of mortality and morbidity are reported worldwide [1]. Therefore, it is time to pay particular attention to this infection.

The first measure to be taken is thorough monitoring of birds. Migratory birds have long been implicated in the spread of avian influenza [2]. They are believed to be infected in Siberia, where they nest, and carry the virus when they travel to Japan to spend the winter. Indeed, it is highly feasible, given that wild birds have been confirmed to be infected in many areas. However, not enough research has been conducted to identify the origin and route of infection of HPAIV. Thorough surveillance of migratory birds is a possible way to prevent the spread of infection and large-scale industrial damage.

HPAIV is also infectious to mammals. Mammal-to-mammal transmission has been reported regularly [3]. In Japan, HPAIV was detected in a fox in April 2022, the first reported case of HPAIV detection in mammals in the country. It is essential to monitor the ecology of as many wildlife species as possible, including crows and mammals directly involved with migratory birds. However, currently available methods, such as long-term field observations and fixed-point cameras, are labor-intensive and time-consuming, making it difficult to monitor such a wide range of ecosystems. Thus, it is necessary to invest the budget for developing new monitoring technologies that utilize artificial intelligence and other technologies to manage the integrated data [4].

Large-scale industrial-level poultry-rearing practices need to be reviewed across the country. Japan's livestock administration has promoted large-scale industrial overcrowding. However, this production system makes livestock vulnerable to infectious diseases and fails to ensure sustainability. Awareness of animal welfare has increased over the recent years, especially in European countries, adding various regulations in farming practices. Limiting the number of animals raised per square foot is such an example [5]. As for Japan, the "Strategy for Sustainable Food Systems, MeaDRI" was established in 2020 to improve productivity and sustainability in the food, agriculture, forestry, and fisheries industries. A budget of 7–8 billion yen is being resourced every year. However, the strategy focuses little on animal welfare and how novel technology can sustain industrial overcrowding. The recent bird flu outbreak and 2010 foot-and-mouth disease has resulted in the culling of more than 210,000 pigs and cattle. It is, therefore, crucial to revise the livestock farming practices across Japan. Switching livestock production policies to flat-raising and pasture-raising in which animals can move freely and eat a natural diet can be a possible solution.

In Japan, the measures taken in response to avian influenza are positioned as an area of "Disaster Response and Recovery Assistance." [1] In this context, it was thankful that the spread of avian influenza occurred during the COVID-19 pandemic. In 2021, the Japanese government's budget for COVID-19 control was approximately 77 trillion yen more than the reconstruction budget for the Great East Japan Earthquake 32 trillion yen over 10 years. While it is relatively easy to gather funding and interest to improve clinical treatment in medicine, infectious diseases involving animals pose a greater risk to humans. However, these concerns can be addressed with the unprecedented government awareness of the infectious disease crisis. Moreover, it is reported that the 10-year funding required to prevent a pandemic is less than one-fifth of the economic loss brought about by the COVID-19 pandemic. The issues that need to be addressed are highly likely to be shared worldwide; therefore, it is essential to promote efforts to prevent a pandemic caused by avian influenza through international collaboration.

## Author's contributions

Conception and designing of the study; KY.

Data collection; OA and TT.

Data analysis and interpretation; KY.

Writing this paper; KY.

Critical revision of the paper; OA, GT and TT.

All the authors read the final draft and approved submission.

## Funding

None.

## Declaration of competing interest

Dr Ozaki reported personal fees from Medical Network Systems Inc. and Kyowa Kirin co. ltd. outside the submitted work. Dr Tanimoto reported personal fees from Medical Network Systems Inc. and Bionics co. ltd., outside the submitted work. No other disclosures were reported.
